# The Virtual Morris Water Task in 64 Patients With Bilateral Vestibulopathy and the Impact of Hearing Status

**DOI:** 10.3389/fneur.2020.00710

**Published:** 2020-08-11

**Authors:** Bieke Dobbels, Griet Mertens, Annick Gilles, Julie Moyaert, Raymond van de Berg, Erik Fransen, Paul Van de Heyning, Vincent Van Rompaey

**Affiliations:** ^1^Faculty of Medicine and Health Sciences, University of Antwerp, Antwerp, Belgium; ^2^Department of Otorhinolaryngology and Head and Neck Surgery, Antwerp University Hospital, Edegem, Belgium; ^3^Department of Otorhinolaryngology and Head and Neck Surgery, Zuyderland Medical Center, Heerlen, Netherlands; ^4^Division of Balance Disorders, Department of Otorhinolaryngology and Head and Neck Surgery, Maastricht University Medical Center, Maastricht, Netherlands; ^5^Faculty of Physics, Tomsk State University, Tomsk, Russia; ^6^StatUa Center for Statistics, University of Antwerp, Antwerp, Belgium

**Keywords:** spatial cognition, vestibular loss, hearing loss, hippocampus, Morris Water Maze

## Abstract

**Background:** Previous studies have demonstrated spatial cognitive deficits in patients with bilateral vestibulopathy (BVP). However, BVP patients frequently present with a concomitant sensorineural hearing loss, which is a well-established risk factor of cognitive impairment and incident dementia. Nonetheless, previous research on spatial cognitive deficits in BVP patients have not taken hearing status into account.

**Objective:** This study aims to compare spatial cognition of BVP patients with healthy controls, with analyses adjusting for hearing status.

**Methods:** Spatial cognition was assessed in 64 BVP patients and 46 healthy controls (HC) by use of the Virtual Morris Water Task (VMWT). All statistical analyses were adjusted for hearing (dys)function, sex, age, education, and computer use.

**Results:** Overall, patients with BVP performed worse on all outcome measures of the VMWT. However, these differences between BVP patients and healthy controls were not statistically significant. Nonetheless, a statistically significant link between sensorineural hearing loss and spatial cognition was observed. The worse the hearing, the longer subjects took to reach the hidden platform in the VMWT. Furthermore, the worse the hearing, the less time was spent by the subjects in the correct platform quadrant during the probe trial of the VMWT.

**Conclusion:** In this study, no difference was found regarding spatial cognition between BVP patients and healthy controls. However, a statistically significant link was observed between sensorineural hearing loss and spatial cognition.

## Introduction

A growing body of literature recognizes that the function of the vestibular system goes far beyond balance and gaze stability. Both animal and human research suggests that the vestibular system plays a critical role in cognition (1–4). According to the Diagnostic and Statistical Manual of Mental Disorders (DSM-V), cognitive functioning can be subdivided into six domains: language, learning and memory, social cognition, attention, executive function, and visuospatial abilities (5). Of these, it seems that visuospatial abilities, which compromises spatial memory and navigation, is by far the most studied cognitive domain in animals and humans with loss of peripheral vestibular input (2, 4, 6). For example, spatial cognition has been studied in patients with vestibular loss using the Virtual Morris Water Task (VMWT) (3, 7, 8). This is a virtual version of the Morris Water Maze, considered the golden standard for assessing spatial cognition in rodents (9). Impaired spatial cognition has repeatedly been observed in patients with bilateral vestibulopathy (BVP) (3, 7). Patients with BVP suffer from a bilateral partial or complete loss of function of the vestibular structures of the inner ear, vestibular nerves, or a combination of both. BVP patients often present with oscillopsia and gait imbalance as primary complaints (10).

The link between spatial cognition and the vestibular system is of clinical importance for several reasons. First, cognitive training might yield therapeutic opportunities for BVP. Conventional treatment for patients with BVP is limited to counseling and intensive daily vestibular physical therapy to improve gaze and postural stabilization (11). However, these therapeutic strategies often remain insufficient (12). Although the utility of cognitive training has been demonstrated to enhance balance in the elderly and in patients with mild cognitive impairment and dementia, cognitive training is not included in the current treatment of BVP (13, 14). According to a recent computational model, cognitive training facilitates the central compensation process in BVP patients by increasing the knowledge about self-motion (15).

Second, interest has been directed toward the link between cognitive impairment and the vestibular system because of the rising prevalence of dementia. As in BVP patients, impaired spatial cognition is among the most frequently observed cognitive deficits in patients with dementia. One of the hallmark symptoms of Alzheimer's disease is wandering behavior and loss of topographic memory (16). The vestibular system, more than any other sensory system, makes widespread cortical projections, including to the hippocampus. The hippocampus is thought to play a key role in the neuronal substrate underlying spatial cognitive deficits in BVP patients (4). For instance, in a leading study by Brandt et al., BVP patients showed bilateral hippocampal atrophy and spatial cognitive deficits (7). Interestingly, in Alzheimer's disease, damage to the hippocampus is the most important anatomopathological feature (17).

Furthermore, several studies have found significantly poorer vestibular function in patients with dementia compared with their healthy peers (18–20).

These observations have led to the hypothesis that vestibular loss might cause cognitive decline and thus may contribute to the development of dementia. Given the rising prevalence of dementia and the lack of curative treatment, the identification of potentially modifiable risk factors is crucial (21).

In the previous literature, however, little attention has been paid to the hearing status of vestibular patients when drawing conclusions about the link between cognitive decline and the vestibular system. A systematic review pointed out that none of the studies investigating cognition in BVP patients have adjusted their analysis for the hearing status of the enrolled subjects (6). However, because of the close anatomical relationship between the vestibular system and the cochlea, hearing loss is observed in up to half of BVP patients (22, 23). Hearing loss is a well-established risk factor for dementia (24–26). Therefore, it is uncertain whether the cognitive deficits observed in BVP patients can be solely attributed to their vestibular loss as previously assumed. The frequently associated hearing loss in BVP patients might also play an essential role in their cognitive impairment (6).

The goal of this study is to compare spatial cognitive performance, assessed using the VMWT, of BVP patients with healthy controls. In contrast to previous studies, the analyses in this study were specifically designed to take the hearing loss of BVP patients into account.

## Methods

### Study Design

The current study was a single-center, prospective, cross-sectional study, recruiting from October 2017 until August 2018 at the Antwerp University Hospital. The study was approved by the local ethics committee of the Antwerp University Hospital/University of Antwerp (protocol number 16/42/426) and informed consent was obtained in all study participants before the start of the study. The study was registered on ClinicalTrials.gov (NCT03690817). The majority of the enrolled participants received general cognitive assessment at another scheduled appointment on a different day. Results have been published earlier (27).

### Study Participants

BVP patients were recruited from the Otorhinolaryngology, Head and Neck Surgery Department at Antwerp University Hospital, Belgium. Inclusion criteria for the BVP group were (1) BVP disease duration of more than 6 months and (2) definite diagnosis of BVP as defined by the diagnostic criteria of the Bárány Society (28):

- Horizontal angular vestibulo-ocular reflex (VOR) gain <0.6 measured by the video head impulse test (vHIT), and/or
- Reduced caloric response (sum of bithermal, 30 and 44°, maximum peak slow phase velocity (SPV) on each side <6°/s), and/or
- Reduced horizontal angular VOR gain <0.1 upon sinusoidal stimulation on a rotatory chair.

Control participants were recruited by means of the population registries at the local city councils in southern Antwerp (Belgium), by advertisements in the hospital, and by approaching friends, family, and colleagues. Only control subjects with no history of vertigo, scores <5 on the Dizziness Handicap Inventory, and normal hearing thresholds at 0.25–8 kHz, based on age and sex (defined by the BS 6951:1988, EN 27029:1991, and ISO 7029-1984 standards), were enrolled in the study.

The following additional inclusion criteria were applied for both BVP patients and healthy controls: (1) age ≥18 years, (2) fluency in Dutch, (3) no history of neurological diseases (e.g., dementia, Parkinson's disease, cerebrovascular accident, etc.), (4) absence of clinical signs indicating dementia or mild cognitive impairment, and (5) normal or appropriate corrected vision.

Regarding the necessity of computer use in the VMWT, all participants were asked about their frequency of computer use (daily vs. 2–5 days/week vs. seldom/never). Education of all participants was categorized as primary school, lower secondary school, upper secondary school, and college/university.

### Vestibular Testing

By enrollment in the study, all BVP patients received new neuro-otological testing on site. The evaluation of the lower and mid frequencies function of the lateral semi-circular canals was performed by electronystagmography with bithermal caloric tests and rotatory chair test (Nystagliner Toennies, Germany). At our clinic, rotatory chair tests are performed using sinusoidal rotation (0.05 Hz) with a peak velocity of 60°/s (29). More detailed methodology and normative data were previously described (29). High-frequency function of all six semi-circular canals was measured by the vHIT. In the standard procedure used at our clinic, 10 valid head impulses are required for each canal. Angular head velocity was determined by three mini-gyroscopes, eye velocity by means of an infrared camera recording the right eye, all incorporated in commercially available vHIT goggles (Otometrics, Taastrup, Denmark). VOR gain was defined as the ratio of the area under the eye velocity curve to the head velocity curve from the impulse onset until the head velocity was again 0 (30).

### The Virtual Morris Water Task (VMWT)

**Figure 1:**
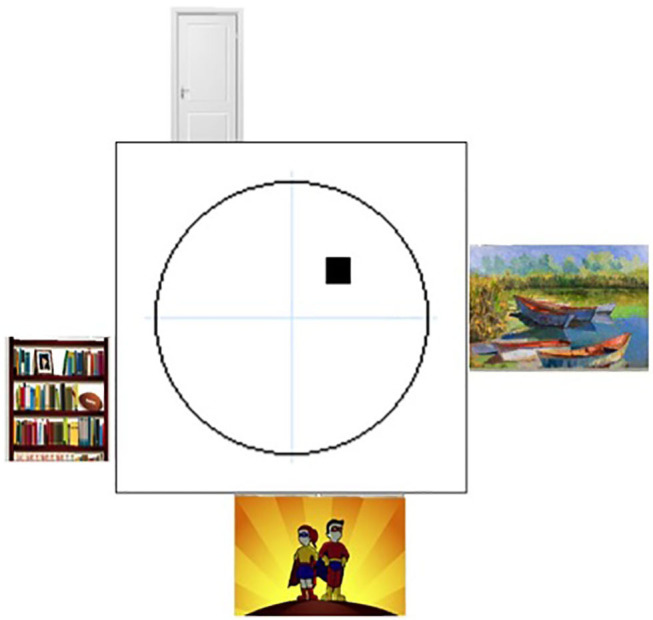
The environment of the Virtual Morris Water Task. Overview of the virtual environment used in the Virtual Morris Water Task, with on each wall a different visual cue. The platform is indicated by the black square in the northeast quadrant of the pool.

To assess spatial learning and spatial memory retrieval, the VMWT was used. This task was designed by Derek Hamilton and was inspired by the original animal research tool, which is considered the gold standard for testing spatial cognition in rodents (9, 31). A 15.6-in. PC laptop monitor was used to display the virtual environment generated by the VMWT software version 1.10 (Neuro Investigations). In this task, participants had to navigate toward a hidden platform as fast as possible. The virtual environment consisted of a round pool, located in the middle of a square room. Each wall of the room contained a different visual cue on which a participant could rely to find his way to the hidden platform. The cues were positioned in such a way that the platform could not be encountered by simply moving toward a single cue (see Figure 1). On the computer screen, a first-person view of the virtual environment was shown. Participants could move in the pool by using the arrow keys on the keyboard. Backward movement or up–down movement was not possible.

Before testing, all participants were given the same written instructions. Afterwards, time to ask questions to the examiner was foreseen. In both groups, four phase trials were performed:

#### Phase I—Exploration Trial

The first part consisted of one block of four trials with a hidden platform. Participants were familiarized with the concept of the game and the use of the key arrows. By observing the participant, the examiner checked for good understanding of the task. When necessary, supplemental explanations were given.

#### Phase II—Hidden Trial

**Figure 2:**
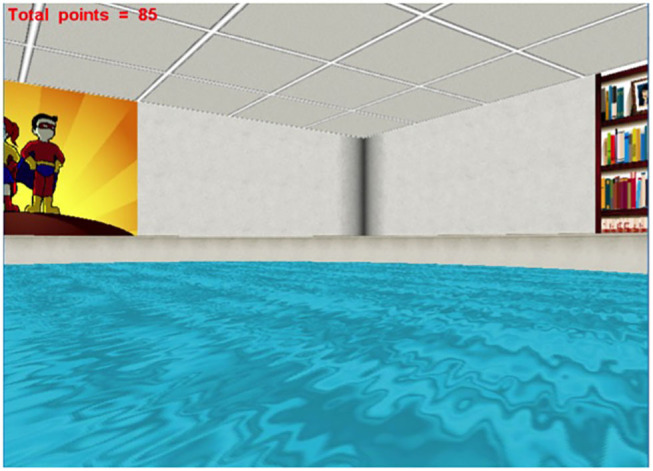
The user's view during the Virtual Morris Water Task. Spatial cognition was assessed by the Virtual Morris Water Task. This figure shows the first-person view of the virtual environment, presented on a computer screen.

**Figure 3:**
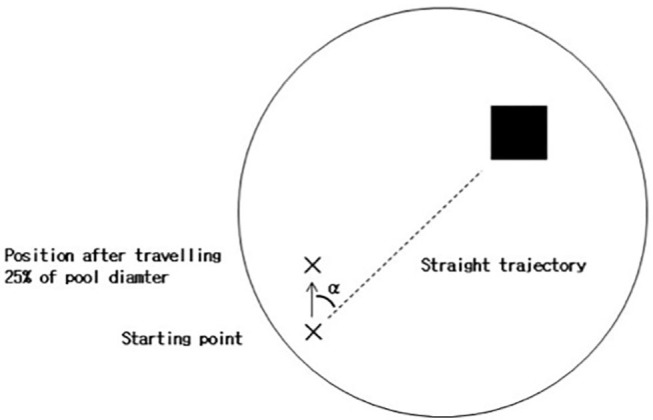
Heading error in the Virtual Morris Water Task. During the hidden trials of the Virtual Morris Water Task, the heading error (α) is computed. When the participant first reaches a distance >25% of the pool diameter from the starting point, the angular deviation from a straight trajectory to the platform is measured. The smaller the heading error, the better the spatial orientation of the participant.

The test was started with 20 hidden platform trials. A virtual environment with different visual cues was used than in the exploration trial (see Figure 2). The hidden platform was located, in all trials, at the same spot in the northeastern quadrant of the pool. As it was submerged underneath the pool's surface, it was not visible to the participants. Starting locations during each trial were sampled pseudo-randomly from the four cardinal direction points of the pool. If participants were unable to find the hidden platform after 60 s, the platform was made visible and a message appeared prompting the participant to swim to the platform. During each of these trials, three measures were computed:

- The latency, i.e., time to reach the platform.
- The covered path length, i.e., total distance traveled, divided by the pool diameter.
- The heading error, when the participant has traveled a distance >25% of the pool diameter from the start position; the angular deviation is computed between the straight trajectory to the center of the platform and the starting position (see Figure 3).

Performance during these hidden platform trials represent a measure for spatial learning performance.

#### Phase III—Probe Trial

Subsequently, the platform was removed from the pool, unbeknownst to the participants. In this one trial, we measured the percentage of time a participant spent in the platform quadrant. A higher percentage was considered to be related to a better spatial memory retrieval of the participant.

#### Phase IV—Visible Trials

The last part of the test represents a control task for motor condition. Participants had to perform eight visible trials in which the platform was visible and participants had to swim to the platform as soon as possible. Again, latency and path length were recorded for each trial.

### Hearing Assessment

To correct cognitive outcome measures for the hearing status of the enrolled participants, a pure tone audiometry was performed. The unaided hearing thresholds were measured in a sound-isolated booth. For air conduction, hearing thresholds were determined at 125, 250, 500, 1,000, 2,000, 3,000, 4,000, 6,000, and 8,000 Hz using a two-channel Interacoustics AC-40 audiometer and insert earphones. Bone conduction thresholds were tested at 250, 500, 1,000, 2,000, 3,000, and 4,000 Hz. The high Fletcher index, which is the mean of air conduction hearing thresholds at 1, 2, and 4 kHz, was calculated for both ears. The hearing status of a participant was defined by the high Fletcher index of the better-hearing ear.

### Data Collection and Statistical Analysis

Data were stored in OpenClinica LLC (Waltham, MA, USA), a secured online database for electronic data registration and data management developed for clinical research. For statistical analyses, IBMS SPSS Statistics (IBM Corp. Released 2016. Version 24.0. Armonk, NY) and “R” was used (R: A language and environment for statistical computing. Released 2013. R Foundation for Statistical Computing, Vienna, Austria).

Depending on distribution, demographic data were analyzed with either *t*-test and χ^2^ test, or Mann–Whitney test and Fisher's exact test. Analogous to previous work, the 20 hidden platform trials were divided into three blocks: block 1 with trials 1–4, block 2 with trials 5–12, and block 3 with trials 13–20. First, for each performance variable of the hidden trial (latency, path length, and heading error), a linear mixed model was fitted. A random effect of individual was added to account for the non-independence between observations from the same individual. Fixed effects included group (BVP vs. healthy controls), time (repeated measurements during the three blocks of trials), and their interaction. The latter interaction term evaluates whether there is a difference in learning between BVP patients and healthy controls during the VMWT. In other words, a statistically significant interaction term points out that during all 20 hidden trials, one group progressively found the platform faster compared with the other group, indicating a better learning curve in this group throughout the test.

In the absence of a significant interaction, linear mixed models were fitted for all performance variables of the hidden trials, with the main factors group (BVP vs. healthy controls) and time (repeated measurements during the three blocks of trials), without interaction term and hearing status as indicated by the high Fletcher index. Using these models, we evaluated whether there was a statistically significant different performance between the two participant's groups, BVP and healthy controls, across all trial blocks. Moreover, using this model, we assessed whether there was a statistically significant main effect of the hearing status on VMWT performance. In all of these models, the following covariates were added: age, sex, computer use, and education.

To compare the spatial memory retrieval during the probe trial, a multiple linear regression model was fitted with the main factors group (BVP vs. healthy controls) and hearing status (high Fletcher index of the better-hearing ear). Again, age, sex, computer use, and education were entered as covariates.

Finally, mean path lengths and latencies during the 10 visible trials were computed and used as dependent variables in a similar multiple linear regression model.

## Results

### Participant Characteristics

**Figure 4:**
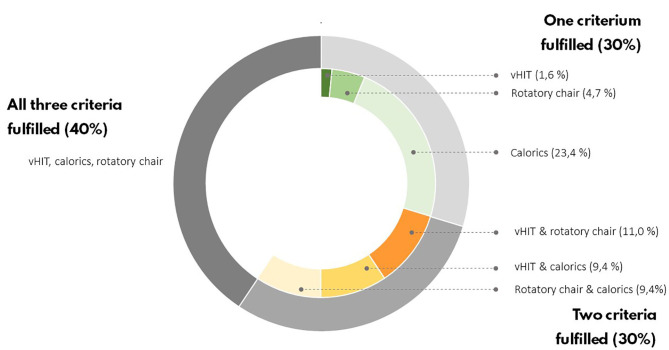
Vestibular test results of BVP patients. According to the Bárány criteria, only one of the vestibular tests have to be bilaterally impaired to establish a BVP diagnosis.

Sixty-four BVP patients with a mean age of 59 ± 14 years met the study inclusion criteria; 60% of them were male. Forty-six healthy controls with a mean age of 48 ± 17 were enrolled in the study; 44% of them were male (Table 1). The BVP group was gender matched to the control group. BVP patients were on average older, less educated, and had less computer experience than healthy controls (*p* < 0.05). Hearing loss was more frequent in BVP patients (High Fletcher index 58 ± 42 dB in BVP patients vs. 11 ± 12 dB in healthy controls, p < 0.05). To diagnose BVP, the Bárány Society criteria needed to be fulfilled (28). Forty percent of BVP patients met all Bárány Society criteria: a bilateral reduced response on caloric testing, rotatory chair test, and vHIT. In 30% of BVP patients, two out of three Bárány Society criteria were fulfilled, and in the remaining 30% of the BVP patients there was only found a vestibular hypofunction in one of the three vestibular tests (Figure 4). The mean gain of the left and right vHIT was, respectively, 4.2 ± 0.3 and 0.47 ± 0.3. The mean gain on the rotatory swing was 0.08 ± 0.08.

**Table 1:** Demographic data.

	**BVP patients**	**Healthy controls**	***P*-value**
	***n* = 64**	***n* = 46**	
Age (mean, SD)	59 (14)	48 (17)	<0.05
Sex (*n*, %)			0.1
Male	38 (60)	20 (32)	
Female	26 (33)	26 (57)	
Years of education (mean, SD)	13 (3)	17 (3)	<0.05
Computer use (*n*, %)			<0.05
Seldom/never	15 (27)	2 (6)	
2–5 days/week	10 (18)	4 (11)	
Daily	31 (55)	30 (83)	
Hearing performance: pure tone audiometry			
Fletcher index better-hearing ear (mean, SD in dB)	58 (34)	11 (12)	<0.05

BVP, bilateral vestibulopathy; dB, decibel.

An underlying cause of vestibular loss could not be identified in 33.9% of BVP patients. With a prevalence of nearly 20%, a mutation in the *COCH* gene causing DFNA9 was the most frequent underlying non-idiopathic etiology in our BVP cohort (35). In 16% of BVP patients, an infectious cause was found (e.g., meningitis, neuritis, Lyme disease). Menière's disease and head trauma accounted for, respectively, 6 and 11% of BVP causes. In four BVP patients, an ototoxic cause was suspected (three aminoglycosides antibiotics and one chemotherapy, not further specified).

### Results of the VMWT

#### Hidden Platform Trials: Spatial Learning

**Figure 5:**
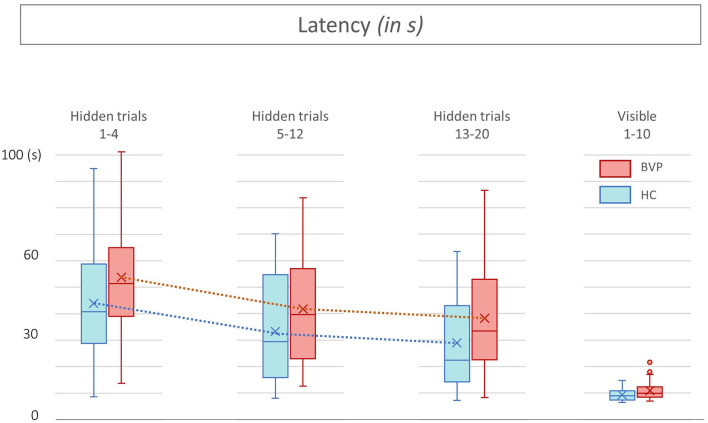
Latencies from patients with bilateral vestibulopathy and healthy controls. This figure shows performance of patients with bilateral vestibulopathy (BVP, red) and healthy controls (HC, blue) during the 20 hidden platform trials and 10 visible platform trials of the Virtual Morris Water Maze. The latency, defined by the time in seconds needed to find the platform, is a measure for spatial learning. During all hidden platform trials, HC outperform BVP patients.

**Figure 6:**
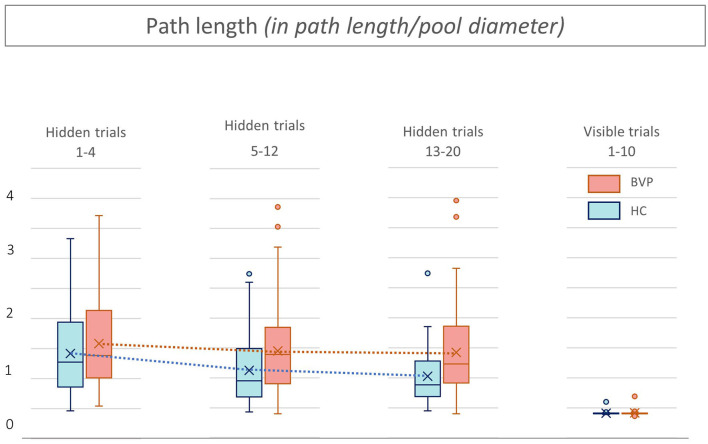
Path length from patients with bilateral vestibulopathy and healthy controls. This figure shows performance of patients with bilateral vestibulopathy (BVP, red) and healthy controls (HC, blue) during the 20 hidden platform trials and 10 visible platform trials of the Virtual Morris Water Maze. The path length, defined by the relative distance to the pool diameter covered to reach the platform, is a measure for spatial learning. During all hidden platform trials, performance of BVP patients is worse than HC.

**Figure 7:**
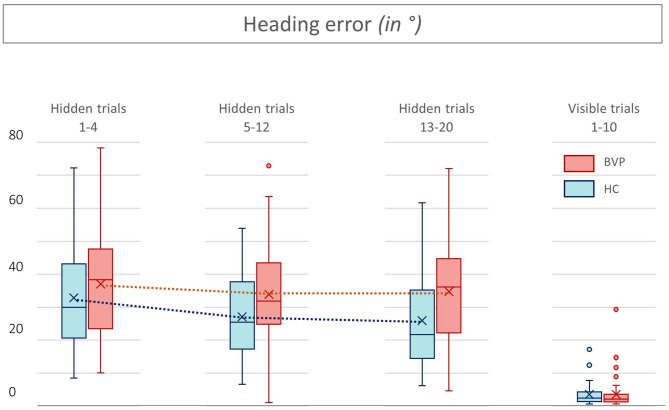
Heading errors from patients with bilateral vestibulopathy and healthy controls. This figure shows the performance of patients with bilateral vestibulopathy (BVP, red) and healthy controls (HC, blue) during the 20 hidden platform trials and 10 visible platform trials of the Virtual Morris Water Maze. The higher the heading error, the worse the performance and thus spatial learning. In all hidden trials, the heading errors of BVP patients are higher than those of HC.

First, during the hidden platform trials, a significant main effect of time was found for all outcome measurements, indicating faster determination of the hidden platform location over time (see Table 2). No statistically significant interaction between group × time was found in any of the outcome measures. This indicates that, regardless of the absolute outcome measurements in both groups, the learning curve in BVP patients was not significantly slower than in healthy controls. This is also illustrated by a similar slope of the curves in BVP patients and controls showed in Figures 5–7.

**Table 2:** Results of the linear mixed models used for the hidden platform trials of the Virtual Morris Water Maze.

***P*-value (effect size if p < 0.05)**	**Group (BVP vs. healthy controls)**	**Hearing status (high Fletcher index of better-hearing ear)**	**Age**
Latency	0.16	0.006 (0.11)	<0.001 (0.86)
Path length	0.28	0.76	0.04 (0.01)
Heading error	0.32	0.56	<0.001 (0.3)

Outcome measures for spatial learning were (1) latency, (2) path length, and (3) heading error. All analyses adjusted for age, sex, computer use, education, and hearing loss. A *p* < 0.05 indicates a statistically significant main effect of the examined factor.

BVP, bilateral vestibulopathy.

Second, as shown in Figures 5, 6, BVP patients took both more time and longer paths, compared with healthy controls, to reach the hidden platform during all three trial blocks (1–4, 5–12, and 13–20). Likewise, the heading error of BVP patients was larger during all three trial blocks (see Figure 7). Importantly, in linear mixed models no significant group effect for latency, path length, or heading error was found. In other words, the worse performance of BVP patients compared with healthy controls was not statistically significant. All these analyses were adjusted for hearing status, age, sex, computer use, and education. Third, a statistically significant association between hearing loss and spatial learning was seen. The higher the Fletcher index, the longer the latencies were during the hidden trials (*p* = 0.006, effect size 0.11). As the Fletcher index increased by 1 dB, the latency was 0.11 second longer. There was no significant effect of hearing loss on path length or heading error.

#### Probe Trial: Spatial Memory Retrieval

During the probe trial, BVP patients searched 38% (±23.3) of their time in the correct quadrant, whereas healthy controls spent 52.1% (±22.7) in the correct quadrant. However, this difference was not statistically significant between the two groups (*p*-value in multiple linear regression model of groups = 0.9).

**Figure 8:**
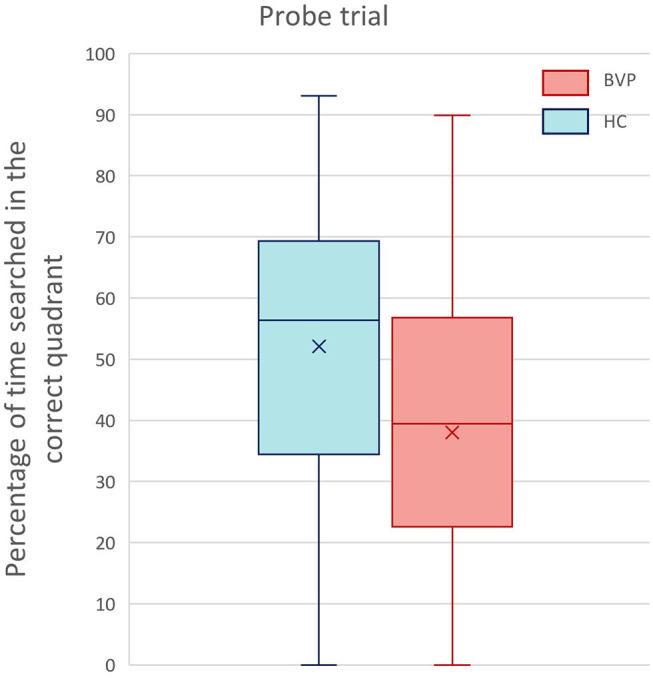
Results of the probe trial of the Virtual Morris Water Task. This boxplot shows the performance of BVP patients (red) and healthy controls (blue) during the probe trial. Spatial memory retrieval was defined by the relative amount of search time (%) spent in the correct quadrant of the pool.

**Figure 9:**
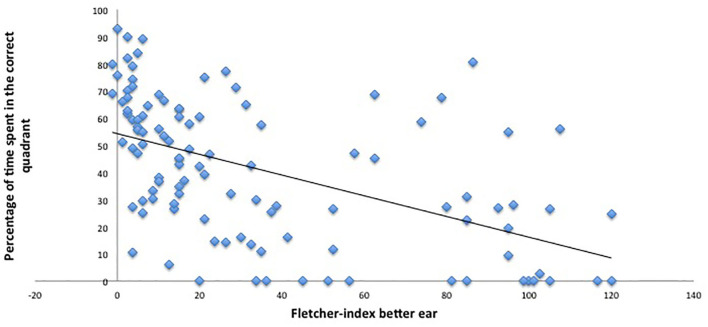
Hearing thresholds and performance in the probe trial. This figure indicates the worse the hearing (higher hearing thresholds or Fletcher indexes), the worse the performance on the VMWT probe trial (less time spent in the correct quadrant).

Nonetheless, the analysis revealed a significant main effect of hearing loss on relative amount of time spent in the correct quadrant (*p* = 0.05, β −0.1). This indicates that the worse the hearing, the poorer the memory retrieval. The results of the probe trial are demonstrated in Figures 8, 9.

#### Visible Trials: Motor Control Condition

No significant effects of group or hearing status were found during the visible platform trials regarding latency, path length, and heading error. This indicates that BVP patients showed no difference in their motor control condition, compared with healthy controls. Results of the visible trials are shown in Figures 5–7.

## Discussion

The present study was designed to evaluate whether BVP patients suffer from spatial cognitive deficits compared with healthy controls. Furthermore, the analyses in this study were especially set out with the aim of evaluating the importance of concomitant hearing loss of BVP patients regarding the suspected relationship between cognition and the vestibular system.

In one of the largest BVP patient group so far, this study found a worse performance on all outcome measures of the VMWT in BVP patients compared with healthy controls. However, this difference was never statistically significant between the BPV group and the healthy control group. In contrast to earlier studies, all statistical analyses of this study included correction for hearing (dys)function. Although no significant group difference was observed, it seemed that, on the other hand, hearing loss was found to be statistically significantly associated with worse spatial cognition. The worse the hearing of BVP patients, the worse the spatial learning indicated by longer latencies in the hidden trial of the VMWT. Likewise, in the probe trial, hearing loss resulted in less time spent in the platform quadrant, which suggests worse spatial memory retrieval.

### Vestibular Loss and Spatial Cognition

Previous studies have not dealt with the hearing status of the enrolled BVP patients when drawing conclusions about the relationship between vestibular loss and cognitive decline (6). Given the observed statistically significant effect of hearing loss on spatial cognition, this study highlights the need to correct for hearing loss when evaluating cognition in vestibular patients. Furthermore, our findings raise intriguing questions regarding the assumed link between cognition and the vestibular system. According to our results, it could be questioned whether the spatial cognitive deficits of BVP patients might be solely attributed to their hearing loss and not to their vestibular loss. However, it is important to bear in mind that the control group in this study included subjects with normal age-appropriate hearing. Hence, some of the control subjects suffered from presbyacusis, but overall the prevalence of hearing loss in the control group is low. Therefore, results should be interpreted with caution and it cannot be concluded that hearing loss is the only factor resulting in the spatial cognitive deficits of BVP patients. Vestibular loss might play an additional role. Moreover, previous studies observing spatial deficits in BVP patients included patients with complete vestibular loss. In this study, patients with BVP, as defined by the Bárány criteria, were included. This implicates that also patients with partial vestibular loss were included, for example, preserved function on vHIT in the absence of caloric function (7). Future work is required to further unravel the link between cognition, vestibular loss, and hearing loss. An interesting study protocol would be to compare spatial cognition between four groups: healthy controls, patients with hearing dysfunction and normal vestibular function, patients with normal hearing and vestibular dysfunction and finally, patients with both vestibular and hearing dysfunction.

Nonetheless, for all future studies investigating cognition in BVP patients, our results implicate that it is obligatory to take the hearing status of BVP patients into account.

### Hearing Loss, Spatial Cognition, and the Hippocampus

In accordance with the present results, previous studies have demonstrated a link between spatial cognition and hearing loss. A recent meta-analysis showed a significant impairment of visuospatial abilities in patients with hearing loss across cross-sectional studies, using a wide variety of spatial cognitive tasks (26).

Two recent animal studies investigated spatial cognition using the Morris Water Maze (36, 37). Mice with presbyacusis were found to have worse spatial learning and spatial memory retrieval compared with mice with normal hearing (36). Likewise, mice with noise-induced hearing loss showed poorer performance during the Morris Water Maze (37). This was pointed out by longer latencies during the hidden platform trials and less time spent in the platform quadrant during a probe trial in mice with noise-induced hearing loss compared with mice with normal hearing. It is important to note that mice typically do not perform well in the Morris Water Maze and authors suggest that they might not use spatial strategy. Hence, caution should be taken to extrapolate these findings to humans (38).

The hippocampus is the area of the brain that has long been implicated in spatial memory. Animal and human studies have shown altered functioning and even atrophy of the hippocampus in subjects with vestibular loss [for review see (4)]. Interestingly, the poorer spatial performance of mice with hearing loss was also accompanied by a decrease of hippocampal neurogenesis (37). As the Morris Water Maze does not rely upon auditory function, authors hypothesize that the auditory input plays a maintenance role for hippocampal function and neurogenesis (37). However, it should be noted that exposing mice to noise trauma does not only result in hearing loss but might also induce peripheral vestibular damage (39, 40). This has not been taken into account in the study. Hence, it is possible that the decreased hippocampal neurogenesis observed in mice with noise-induced hearing loss is (partially) related to a loss of peripheral vestibular input. Vice versa, despite the extensive previous research, many questions remain about the neuroanatomical substrate underlying the association between the vestibular system, spatial cognition, and the hippocampus. Little research has been carried out to investigate if subjects with hearing loss have altered hippocampal function and volume. Hence, it could conceivably be hypothesized that hearing loss plays a role in the assumed neuroanatomical pathways between the peripheral vestibular input and the hippocampal and cortical areas involved in spatial cognition.

### The VMWT to Assess Spatial Cognition in BVP Patients

In previous literature, the VMWT seemed to be one of the most used tools to assess spatial cognition in vestibular patients (2). In patients with complete loss of vestibular input after a bilateral vestibular neurectomy, distinct poorer performance was observed on the hidden and probe trials of the VMWT (7). In a more recent study of the same group, patients with severe but incomplete BVP showed more subtle spatial cognitive deficits (3). Likewise, in patients with a unilateral loss of vestibular input, only one of the outcome measures was impaired in patients with right unilateral vestibulopathy. In the patients with left unilateral vestibulopathy, none of the outcome measures differed significantly from controls (8). These results suggest that with increasing loss of peripheral vestibular input, the spatial cognition decreases. In our study, most BVP patients suffered from a deep but incomplete loss of vestibular function. It is possible, therefore, that the worse VMWT performance in our BVP patients did not yield statistically significance level.

Furthermore, the purely stationary set-up of the VMWT might underestimate the real-life spatial cognitive deficits of BVP patients as a result of loss of vestibular input. Previous research has established that, while navigating, an “inner neural map” is created, based on peripheral vestibular input. This neural representation of the external environment is computed in the hippocampus and entorhinal cortex and consists of several cooperative cell types: angular head velocity cells; head direction cells; place and grid cells (41). Rodent studies have demonstrated that vestibular input modulates the activity of the head direction cells and the place cells (33, 42). As the VMWT is static, the task does not rely on any vestibular input from real locomotion. Hence, it is likely that real-life navigation tasks will be more sensitive to reveal spatial cognitive deficits in BVP patients.

Moreover, attentional deficits are demonstrated in BVP patients (3, 6, 43). According to Kahneman's Capacity Model of Attention in which an individual has a limited total amount of cognitive resources available to divide among mental tasks, dual tasking might be more demanding in BVP patients because of the increased attentional need for keeping balance (34). As subjects stay seated during the VMWT, attentional resources can be fully directed toward the spatial memory task. This might be a second reason why the VMWT underestimates the real-life spatial cognitive deficits of BVP patients.

To sum up, the VMWT is a widely used method to assess spatial cognition in humans (9). Several studies have demonstrated spatial cognitive deficits in BVP patients, using the VMWT (3, 7, 8). However, previous studies have not dealt with the concomitant hearing loss of BVP patients. As hearing loss is a risk factor for dementia, this might be an important forgotten factor. This is the first study investigating spatial cognition by use of the VMWT in a BVP group as large as 64 patients, and with correction for the hearing (dys)function in all analyses. All outcome measures of the VMWT were worse in BVP patients compared with healthy controls; however, these differences were not statistically significant. Contrarily, hearing loss was statistically significantly associated with worse spatial learning and spatial memory retrieval. Regarding our study protocol with healthy controls without severe hearing loss, it is not excluded that vestibular loss has an additional effect on spatial cognition. Nonetheless, our findings confirm the negative repercussion of hearing loss on spatial cognition (26), and highlight the need to correct for hearing loss when investigating cognition in a vestibular population group.

Both vestibular and hearing dysfunction are prevalent in the elderly (32, 44, 45). Given the rising prevalence of dementia, and the current lack of therapy, future studies are needed to identify modifiable risk factors (16). Therefore, the link between cognitive decline and the hippocampus on the one hand and hearing loss and vestibular loss on the other hand needs to be further unraveled. To develop a full picture, a study protocol that would additionally include patients with normal vestibular function but different levels of sensorineural hearing loss would be interesting. Furthermore, considering the static and single task paradigm involved in the VMWT, real navigation tasks might give more insights in the potential spatial cognitive deficits related to the loss of vestibular input.

### Limitations

The subjects in our HC group were not perfectly matched to BVP patients regarding age, education, and computer experience. As HC were on average younger, more educated, and more computer experienced, VMWT performance could be relatively overestimated in the HC group. Regarding the observed negative effect of hearing loss on spatial cognition (longer latencies and less time spent in the correct quadrant), it is important to bear in mind that the majority of patients with hearing loss were in the BVP group. Therefore, it is not excluded that vestibular loss plays an additional role in spatial cognition, which could not be observed in this study protocol using healthy controls with normal hearing. Second, there was a correlation with hearing loss and age. Although all models were corrected for age, it is not excluded that age might play an important role in the observed link between spatial cognition and hearing loss.

## Conclusion

The present study assesses spatial cognitive performance in one of the largest BVP cohorts so far. The study was especially designed to determine the relative importance of hearing loss in spatial cognition of BVP patients, as this has been frequently overlooked. We found worse spatial cognitive performance on all outcome measures of BVP patients. However, these differences were not statistically significant between the BVP patients and healthy controls, when corrected for age, gender, education, level of computer use, and hearing loss. Interestingly, only hearing loss was found to be statistically significantly associated with worse spatial cognition. These findings highlight the need to correct for hearing loss in future studies investigating cognition in BVP patients. As the control group did not include subjects with severe hearing loss, an additional effect of vestibular loss on spatial cognitive performance cannot be excluded.

## Additional Comments

These data have partially been presented on the 30th Bárány conference, Uppsala, June 2018.

## Data Availability Statement

The raw data supporting the conclusions of this article will be made available by the authors, without undue reservation.

## Ethics Statement

The studies involving human participants were reviewed and approved by local ethics committee of the Antwerp University Hospital/University of Antwerp (protocol number 16/42/426). The patients/participants provided their written informed consent to participate in this study.

## Author Contributions

BD: data collection, statistical analyses, study concept, and writing manuscript. GM, RB, and PV: study concept and supervision. JM: data collection. EF: study concept and statistical analyses. VV: study concept, writing manuscript, and supervision. All authors contributed to the article and approved the submitted version.

## Conflict of Interest

The authors declare that the research was conducted in the absence of any commercial or financial relationships that could be construed as a potential conflict of interest.
